# Piloting Rasch model scoring of the National Eye Institute Visual Function Questionnaire in uveitis

**DOI:** 10.1186/s12348-024-00398-x

**Published:** 2024-04-16

**Authors:** Lisa Gittel, Jeany Q. Li, Jennifer Dell, Maximilian W. M. Wintergerst, Carsten Heinz, Robert P. Finger, Jan Henrik Terheyden

**Affiliations:** 1 Department of Ophthalmology, University Hospital Bonn, Venusberg-Campus 1, Bonn, NRW 53127 Germany; 2 Department of Ophthalmology, Faculty of Medicine and University Hospital of Cologne, University of Cologne, Cologne, Germany; 3 Department of Ophthalmology, St. Franziskus Hospital Münster, Münster, Germany; 4 Department of Ophthalmology, University Duisburg-Essen, Essen, Germany; 5 Department of Ophthalmology, University Hospital Mannheim & Medical Faculty Mannheim, University of Heidelberg, Mannheim, Germany

**Keywords:** Uveitis, Quality of life, Patient-reported outcomes

## Abstract

**Introduction:**

The National Eye Institute Visual Function Questionnaire (NEI VFQ) is a common patient-reported outcome measure (PROM) in uveitis trials. Its psychometric properties using state-of-the-art scoring based on Rasch models, a latent trait model that improves accuracy of PROMs assessment, has not yet been investigated.

**Methods:**

The study participants were recruited online from uveitis patient organizations, where individuals self-reported their uveitis diagnosis and visual acuity level. These participants then completed the NEI VFQ-25. The visual function (VF) and socioemotional (SE) subscales were psychometrically analysed in terms of item fit, targeting, internal consistency, dimensionality, and differential item functioning (DIF), using Rasch models. Criterion validity was examined based on associations between NEI VFQ person measures and recent visual acuity (VA) levels.

**Results:**

Ninety-nine participants recruited online from uveitis patient organizations (68 women, 31 men; mean age 50 ± 15 years; 46.5% self-reported receiving systematic therapy for uveitis, 0.6% NEI VFQ-25 missing data) were included. The mean difficulty of items was lower than the average person ability. None of the items demonstrated misfit to an extent that would induce noise into the measurement. The consistency metrics person reliability and person separation index of the subscales were 0.85 and 2.34 (NEI VFQ-VF), 0.86 and 2.52 (NEI VFQ-SE), respectively. There was no evidence of multidimensionality and none of the items showed DIF by gender. The differences between item and person measures were 1.44 (NEI VFQ-VF) and 1.03 (NEI VFQ-SE). NEI VFQ-25 person measures were significantly lower in participants with visual impairment (all *p* values ≤ 0.007).

**Conclusion:**

Rasch model-based scoring of the re-engineered NEI VFQ-25 demonstrates acceptable internal consistency, item fit and construct validity for assessing two key domains of quality of life in individuals self-reporting uveitis. The PROM was targeted at a higher level of difficulty than present in our heterogeneous sample.

**Supplementary Information:**

The online version contains supplementary material available at 10.1186/s12348-024-00398-x.

## Introduction

Uveitis can have detrimental effects on visual function and quality of life (QoL) in affected people and can have serious complications leading to blindness and long-term disability [1–3]. Patient-reported outcome measures (PROMs) are commonly used to assess QoL, and they become increasingly integrated into regulatory drug approval processes and routine clinical practice in the context of uveitis [4].

The National Eye Institute Visual Function Questionnaire (NEI VFQ) is among the most frequently used PROMs in ophthalmology [1, 5, 6] and assesses vision-related QoL (VR-QoL). It was developed based on a literature review, focus group discussion with patients and expert panel input, covering five eye conditions (cataracts, glaucoma, age-related macular degeneration, diabetic retinopathy, cytomegalovirus (CMV) retinitis) [7]. The most common version of the NEI VFQ includes 25 items spanning eleven vision-related domains, while other versions with e.g. 51 and 39 items are available [8–10].

The NEI VFQ is also a commonly used PROM in uveitis trials [1, 2, 4–6, 11, 12]. Its internal consistency, test–retest reliability, reproducibility, and convergent validity in uveitis has been supported by previous psychometric analysis but this was based on conventional sum scoring [13]. However, this scoring of the NEI VFQ comes with problems in the stability of the measured construct [6], and use of a scoring system based on latent trait models has been recommended more recently [14, 15]. The lack of use of these modern scoring methods in uveitis have been recently criticized [4].

In this study, we have addressed this by evaluating the psychometric properties of the NEI VFQ-25 in uveitis patients. We have performed a psychometric analysis of the NEI VFQ-25 [6] using the primary items and the subscale structure of the NEI VFQ-25C [13] based on the Rasch model, a latent trait model, and investigated additional psychometric dimensions of the questionnaire in uveitis patients that cannot be investigated based on the conventional sum scoring system.

## Materials and methods

### Participants

Members of the German uveitis patient organizations Uveitis e.V. and the German Uveitis work group (Deutsche Uveitis Arbeitsgemeinschaft e.V.; DUAG) were recruited for remote participation in this study. The selection of participants was included based on self-reports and included the administration of other sociodemographic and patient-reported outcome questionnaires as well as self-reports of recent best corrected visual acuity (BCVA) data in the better eye, via an online form. The main outcome of the study was outside the work presented here (unpublished data). Inclusion criteria were a reported history of uveitis and available data on the NEI VFQ-25, participants with a high proportion of missing data (more than 50% of items per subscale) were excluded.

The study adhered to the tenets of the Declaration of Helsinki. Since the survey was performed anonymously, the ethics committee at the University Hospital Bonn, Germany waived the requirement of specific ethics committee approval.

### National Eye Institute Visual Function Questionnaire

The 25-item version of the NEI VFQ covers eleven vision-related subscales as per conventional sum scoring, as well as one general health item. It has been used among various ophthalmic conditions [1, 9, 15, 16] but has severe psychometric use limitations when the conventional scoring algorithm is applied, including item fit and dimensionality of the subscales, which leads to imprecise measurements [6]. The application of latent trait models, of which the Rasch model is an example implemented commonly, is state of the art today, which has been used to psychometrically reconstruct the NEI VFQ as a two-dimensional scale (NEI VFQ-25C), including a visual function (NEI VFQ-VF) and socioemotional (NEI VFQ-SE) subscale [14, 15]. Latent trait models assume that the single items of a scale form a common construct and thus increase the precision of measurements and decrease the impact of missing data on the outcome [6, 17]. In Rasch analysis, the probability of a person given a certain response to an item is determined by both the person’s ability (e.g. in visual tasks) and the item’s difficulty. The Rasch model assumes that these are measured on the same underlying scale and that the probability of a particular response can be modelled using a logistic function. By transforming ordinal data into pseudo interval-level scales (expressed in logits), it is possible to accurately compare individuals’ abilities and item difficulties [18–20].

### Psychometric and statistical analysis

We investigated the psychometric properties of the NEI VFQ-25 in a uveitis cohort based on Rasch models, using the primary items of the NEI VFQ-25C [15]. We generated a person-item map to visualize the difficulty of test items in relation to the abilities of individuals and further evaluated the targeting of the scale based on the difference between mean item and person measures [21]. We assessed item fit, using infit mean-square (MNSQ) and outfit MNSQ values. Values within the range of 0.5 to 1.5 were considered indicative of effective measurement [22]. Internal consistency was analysed based on the metrics person reliability (PR) and person separation index (PSI), where values above 0.8 and 2.0 were considered acceptable, respectively [22]. The dimensionality of the pre-established subscales was investigated based on a principal component analysis (PCA) of the model residuals. Lastly, we examined DIF for the participants’ sex. A significant standard threshold of > 1 logit units was used as an indicator of DIF.

Person measures were compared between groups with binocular visual impairment (VI), monocular VI, and no VI [23], using the Kruskal–Wallis test and a post-hoc Mann–Whitney-U-test. Rasch analysis was conducted with Winsteps software (version 3.92.1, Chicago, IL). Statistical analysis was performed using IBM SPSS, versions 25 (IBM, Armonk, NY) and *p* values < 0.05 were considered statistically significant.

## Results

A total of 107 responses were available. After excluding participants with > 50% missing responses in either of the subscales, ninety-nine participants (68 females, 69%; 31 males, 31%) were included in our analysis. In the final dataset, 13 item responses to the NEI VFQ were missing (0.6% of all responses). The mean age at participation was 49.6 ± 14.6 years. Sixty-one (61.6%) participants indicated having an occupation whereas thirty-eight (38.4%) indicated being unemployed. Forty-six participants (46.5%) received systemic immunomodulatory therapy. The mean BCVA of the better eye was 0.17 ± 0.28 logMAR units and 0.51 ± 0.62 logMAR units in the worse eye (data available in 79 participants, 80%). Seven participants (9%) had binocular VI, 24 participants (30%) had monocular VI, and 48 participants (61%) had no VI [23].

**Fig. 1:**
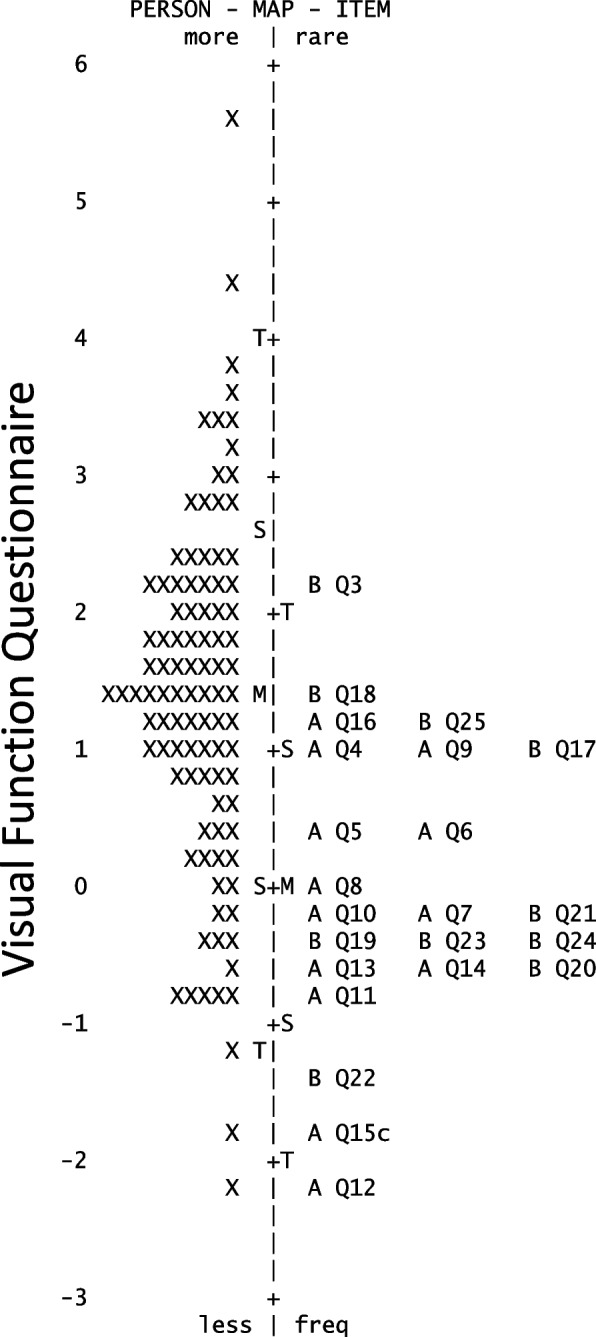
Person-item map of the National Eye Institute Visual Function Questionnaire in the uveitis sample. Items marked with an A belong to the visual function subscale, and items marked with a B are part of the socioemotional subscale. Individual respondents are shown on the left and items are displayed on the right. Items are plotted according to their order of difficulty with the easiest at the bottom and the most difficult at the top of the Figure

The person-item map of both NEI VFQ subscales, NEI VFQ-VF and NEI VFQ-SE, revealed a lower average item difficulty than the average person ability, meaning that the items of the instrument were, in general, too easy for the ability level of the participant (Fig. 1).

Initially, three items of the visual functioning subscale (item 4 [pain], infit mean square 1.81 and outfit mean square 2.03; item 6 [work up close], infit mean square 0.46; item 7 [finding objects on crowded shelf], infit mean square 0.48) and one item of the socioemotional subscale (item 3 [worry about eyesight], outfit mean square 1.78) misfit the Rasch model, suggesting that these items did not effectively measure the underlying construct and introduced noise into the measurement. To address this, we removed 15 misfitting responses from the visual functioning subscale and excluded 23 misfitting responses from the socioemotional subscale for the psychometric investigation. This adjustment improved the fit statistic but did not resolve the misfit of item 6 (infit mean square 0.46) of the visual functioning subscale (Supplementary Figure). Person measures before and after removal of misfitting responses were positively correlated (*r* = 0.99 [0.99–1.00]).

The PR and the PSI fell within the recommended range (Table 1). The NEI VFQ-VF subscale was targeted at a lower functioning level than that of the participants. PCA indicated potential multidimensionality of the NEI VFQ-SE subscale within the cohort, but an exploration of the contrasting clusters indicated a high correlation between the respective person measures (*r* = 0.95 [0.92, 0.96]), supporting unidimensionality of the socioemotional subscale of the NEI VFQ. No significant DIF by sex was observed in any of the NEI VFQ items.

**Table 1:** Psychometric characteristics of NEI VFQ questionnaire in the sample, compared with the Rasch model requirements

	Rasch model requirements	NEI VFQ	
		visual function	socioemotional
Misfitting items	0	**1**^a^	0
PSI	> 2.0	2.34	2.52
PR	> 0.8	0.85	0.86
Cronbach’s alpha	n/a	0.96	0.93
Targeting	< 1 (< 2)	1.44	1.03
Unexplained variance in PCA	< 2.0	1.83	**2.48**

Deviations from literature recommendations are marked in bold

*n/a* Not applicable, *PCA* Principal component analysis, *PSI* Person separation index, *PR* Person reliability

^a^The misfitting item was retained as its MNSQ value fell below a level that would compromise the measurement system

**Fig. 2:**
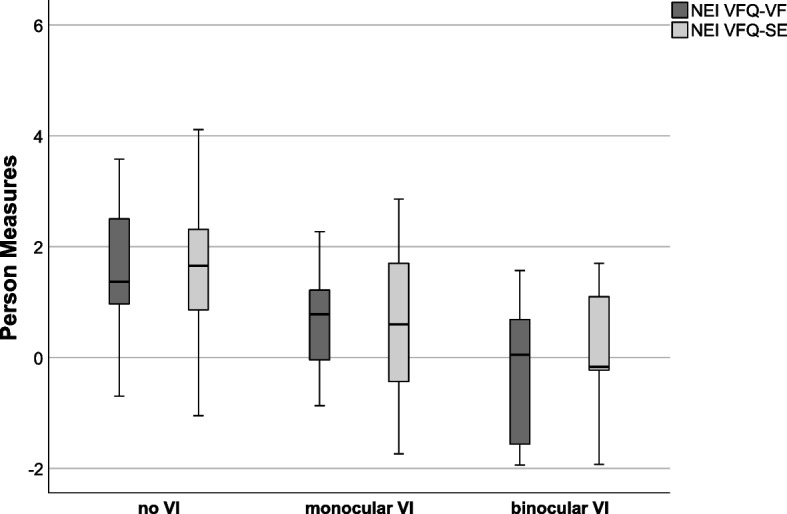
Person measures of the NEI VFQ-VF and NEI VFQ-SE were compared between groups with binocular VI, monocular VI, and no VI using the Kruskal–Wallis test. NEI VFQ = National Eye Institute Visual Function Questionnaire; SE = socioemotional; VF = visual function; VI = visual impairment

VR-QoL was significantly different across VA levels (all *p* values ≤ 0.003, Kruskal–Wallis-test). Post-hoc testing revealed lower VRQoL in individuals with binocular or monocular VI, compared to participants with no VI (all *p* values ≤ 0.007, U-test; Fig. 2).

## Discussion

Our data support the item fit, internal consistency, unidimensionality and construct validity of the NEI VFQ in a heterogeneous cohort of uveitis patients. This supports that Rasch scoring of the NEI VFQ-25 should be implemented in future uveitis trials, given validity in the investigated sub-populations (e.g. by anatomic location, inflammatory activity, complications).

This study confirms the psychometric findings obtained by methods from classical test theory [13]. Traditional sum scoring indicated a Cronbach’s alpha between 0.87 to 0.94 [13], while alpha was > 0.9 in our study. While previous assessments of psychometric properties (test–retest reliability, reproducibility, convergent validity) of the NEI VFQ in uveitis relied on conventional sum scoring [13], our current study delves further into psychometric dimensions such as item fit, targeting, and DIF, aspects that are not ascertainable through classical test theory methods. Additionally, the analyses reveal adjustments that may further optimize the assessment of VR-QoL in uveitis cohorts, which pends further evaluation.

Our study findings reveal relative mistargeting of the NEI VFQ-VF subscale in a heterogeneous population of uveitis patients, suggesting that the instrument may not effectively capture the full spectrum of VR-QoL issues experienced by uveitis patients. This may relate to the high levels of visual acuity observed in our cohort (mean visual acuity was 0.17 ± 0.28 logMAR units in the better eye). Clinical trials in uveitis often include participants with lower visual acuity levels than those in our study, while visual acuity levels in our cohort were at the upper end of the spectrum expected in uveitis trial populations [2, 11, 12]. Thus, we expect the NEI VFQ-25 to be better targeted at lower visual acuity populations. However, this needs to be further validated in an independent cohort.

Since the analyses were focused on the psychometric properties of scoring method based on Rasch models, our results do not allow commenting on the content validity of the NEI VFQ-25 in uveitis. The development of content domains and validation included 17/246 (7%) individuals with CMV retinitis, an infectious posterior uveitis [7]. The initial validation study of the NEI VFQ (51-item version) also included a proportion of individuals with CMV retinitis (37/598, 6%) [8]. This supports the content validity of the NEI VFQ in a specific uveitis entity but does not allow making assumptions about its validity in other types of uveitis (e.g. non-infectious forms, anterior uveitis), where more research is needed. However, the recent development of the Rasch model-scored version of the NEI VFQ (NEI VFQ-25C [15]), did not report inclusion of any uveitis patients and therefore, our results strengthen the use of an model-based scoring system of the NEI VFQ in instances where the use context justifies including NEI VFQ items to assess of VR-QoL in uveitis.

We have conducted an analysis of psychometric properties of a commonly used PROM in uveitis trials. While our findings suggest overall sound psychometric properties, it is important to acknowledge several limitations. Our sample was recruited via an online survey from patient organizations where only self-reported acuity data and no further clinical data were available, and a reporting bias cannot be fully excluded. As no information on the anatomic classification uveitis or level of inflammation were available, the external validity of our findings may be limited and further research is needed to validate the model-based scoring approach in clinical sub-populations. The performance on the NEI VFQ-25 may vary across different uveitis subtypes as uveitis is a largely heterogeneous condition with diverse clinical presentations and treatment responses. One of the NEI VFQ-25C sub-items (item 16a) could not be included in the analysis but has high content similarity with one item included (item 16), thus we do not expect this to impact the validity of the results. Moreover, the focus group discussions conducted during the development of the NEI VFQ involved only a limited number of uveitis patients. Despite the NEI VFQ-25 being commonly used in uveitis trials, further research is needed to investigate the content validity and patient-reported dimensions that are additionally relevant. Our study sample was recruited form uveitis patient organizations in Germany, possibly limiting its representativeness of uveitis patients internationally.

To address these limitations, future research should incorporate larger, more diverse uveitis populations with verified diagnoses and detailed subtype information, while also considering comprehensive validity assessments (concurrent, convergent, discriminant and known group) to confirm and extend our findings. A broader range of items with varying levels of difficulty and covering different aspects relevant to uveitis patients may be required to fully capture VR-QoL in uveitis.

Overall, the data from our exploratory study support the use of a Rasch model-based scoring algorithm in uveitis patients in the future, which can make PROM assessments more precise. Our study endorses the further use of patient-relevant endpoints in clinical studies in uveitis. Nevertheless, addressing the constraints identified in our research warrants further inquiry in future studies.

### Supplementary Information

**Supplementary Material 1.**

## Data Availability

The data that support the findings of this study are available from the corresponding author on reasonable request.

## Abbreviations

BCVA: Best corrected visual acuity
DIF: Differential item functioning
MNSQ: Mean square
NEI VFQ: National Eye Institute Visual Function Questionnaire
PCA: Principal component analysis
PR: Person reliability
PROMs: Patient-reported outcome measures
PSI: Person separation index
QoL: Quality of life
SE: Socioemotional
VA: Visual acuity
VF: Visual function
VI: Visual impairment
VR-QOL: Vision-related quality of life
