# Supplementary material for: Piloting Rasch model scoring of the National Eye Institute Visual Function Questionnaire in uveitis
Source: J Ophthalmic Inflamm Infect. 2024 Apr 16;14:16. doi: 10.1186/s12348-024-00398-x (PMC11021379; doi:10.1186/s12348-024-00398-x)
Supplement: Supplementary file 1 — Supplementary Material 1. [file 12348_2024_398_MOESM1_ESM.docx]

**Supplementary material**

**FIGURES**

**Supplementary Figure.** Parameter-level mean square item fit statistics of the adjusted National Eye Institute Visual Function Questionnaire 25 in the uveitis sample.
